# Onset Age and Internalizing Problems in Adolescents with Gender Dysphoria: Is There an Association?

**DOI:** 10.1007/s10508-025-03118-1

**Published:** 2025-03-24

**Authors:** Saskia Fahrenkrug, Inga Becker-Hebly, Lena Herrmann, Claus Barkmann, Sarah Hohmann, Carola Bindt

**Affiliations:** Department of Child and Adolescent Psychiatry, Psychotherapy, and Psychosomatics, University Medical Center Hamburg-Eppendorf, Martinistraße 52, W35, 20251 Hamburg, Germany

**Keywords:** Gender dysphoria, Adolescent, Onset age, Internalizing problems, Transgender, DSM-5

## Abstract

An increasing heterogeneity of clinical presentations and varying levels of psychological problems characterize gender dysphoria (GD) in adolescents. These clinical patterns suggest distinct developmental trajectories. Here, we examine the onset age of GD, i.e., the percentage of early onset (EO) vs. late onset (LO), and its association with internalizing problems in adolescents with GD. The sample consisted of 462 adolescents (11–18 years, *M*_age_ = 15.46 years; 392 birth-assigned females, 70 birth-assigned males) who attended the Hamburg Gender Identity Service for Children and Adolescents (Hamburg GIS) in Germany between 2013 and 2021. Onset age was self-reported during clinical interviews and then later scored by clinicians using a DSM-5 rating sheet. When adolescents retrospectively met criteria A and B for childhood-onset GD, they were rated as having an EO. Those who fulfilled neither criteria A nor B in childhood were considered to have a LO. Internalizing problems were assessed using the Youth Self-Report. Overall, 51% (*n* = 237) of adolescents with GD presented with an EO and 49% (*n* = 225) reported diagnostic criteria related to a LO. More than half of the sample (58%, *n* = 266) fell within the clinical range for internalizing problems. Furthermore, LO (as opposed to EO) was significantly associated with reporting more internalizing problems. Our findings emphasize that adolescents with LO represent a particularly vulnerable group whose needs should be considered more closely diagnostically and treatment-wise. A protocol-based approach to the indication of physical interventions may not adequately address current clinical presentations and should be complemented by a differential approach based on individual adolescent development.

## Introduction

The current controversial debate among clinicians and researchers about gender dysphoria (GD) in adolescence is based on observations that raise many questions: Worldwide, specialized centers are showing increasing rates of youth, with a presentation of predominantly birth-assigned female adolescents who identify as boys/transmasculine and often wish for gender-affirming medical interventions (Aitken et al., 2015; Chen et al., 2016; de Graaf et al., 2018, 2021; Herrmann et al., 2022; Kaltiala-Heino et al., 2015; Levitan et al., 2019), or who identify as nonbinary, beyond the established gender dualism of male or female (Chew et al., 2020; Herrmann et al., 2023a, b). Similarly, there have been reports of clinical subgroups of adolescents who, after gender-conforming development in childhood, first present with GD in adolescence (Hutchinson et al., 2020; Zucker, 2019), raising questions about whether these clusters may be different from earlier cases (often with prepubertal onset of GD), for example, regarding their levels of psychological problems (Abbruzzese et al., 2023).

In light of rising treatment numbers worldwide (Thompson et al., 2022; Zhang et al., 2020), attempts are being made to identify distinct developmental pathways in an increasingly heterogeneous patients: In addition to the so-called “rapid-onset GD (ROGD)” phenomenon (Littman, 2018), which describes a presumably “sudden” onset of GD without any previously recognizable clues, other differentiations based on the duration of GD and age at first presentation (Arnoldussen et al., 2023; de Rooy et al., 2024; Sorbara et al., 2020) are also gaining attention. Similarly, it can be useful to differentiate based on onset age, which describes a model (Person & Ovesey, 1974) for the early or late onset of GD criteria during childhood (before puberty) and during or after adolescence. As recently critically discussed by Abbruzzese et al. (2023), onset age has gained additional relevance because previous treatment and follow-up data, which served as evidence to support recommendations for the early use of gender-affirming medical interventions, may not be entirely applicable to the current clientele seen in specialized gender identity services who more and more seem to present during or after the onset of puberty.

The effort to differentiate more recent developmental trajectories illustrates the ethical dilemma for practitioners, who must balance their concern about possible false indications and the thus unclear detransition rates (Cohn, 2023) with the simultaneous attempts to minimize the distress of those affected by persistent GD through gender-affirming medical interventions, as recommended, for example, by the current Standards of Care (SOC-8) of the World Professional Association for Transgender Health (Coleman et al., 2022). A close look at newer developmental pathways in the heterogeneous spectrum of self-definitions can help to enable differentiated and individualized treatment planning and thereby guarantee safe indications for the appropriate treatment.

Most adolescents who attend specialized gender identity clinics express the desire for gender-affirming medical interventions, in the sense of hormone substitution. A distinction must be made between explicit prerequisites, such as a clinical diagnosis of GD (i.e., distress at a perceived discrepancy between one’s gender identity and physical sex characteristics or birth-assigned sex) according to the fifth edition of the *Diagnostic and Statistical Manual of Mental Disorders* (DSM-5) (American Psychiatric Association [APA], 2013) or the tenth edition of the *International Classification of Diseases* (ICD-10) (World Health Organization, 2019), and implicit prerequisites for an indication (e.g., maturity of the adolescent, discussion of other nonmedical options), as formulated by the SOC-8 (Coleman et al., 2022) or established treatment protocols (de Vries et al., 2012, 2014). An approach based on the “Dutch Protocol” (Cohen-Kettenis & Klink, 2015; Delemarre-van de Waal & Cohen-Kettenis, 2006) has been established in Europe in the last 20 years. This protocol is used to formulate strict entry criteria, such as a retrospective “opposite-sex” identification going back to childhood (i.e., prepuberty), also known as early onset (EO) course (de Vries et al., 2011, 2012). The result was a homogeneous treatment group (with relatively few psychological problems, an EO, and a strong cross-gender identification), which was considered eligible for treatment. This group was the first to receive gender-affirming medical interventions, which were then subsequently also assessed in several follow-up studies (de Vries et al., 2011, 2014). Contrary, in the absence of an EO and co-occurring psychological problems, adolescents were not considered eligible for receiving gender-affirming medical interventions (de Vries et al., 2012).

While mid- to long-term follow-up studies of the Dutch cohort showed a clear improvement in the psychological well-being of the treatment group (de Vries et al., 2014), the same improvement could not be demonstrated as clearly in a more heterogeneous sample from our short-term study from Germany (Becker-Hebly et al., 2021) that also included late onset (LO) courses (20% of the sample). Similarly, a short-term study of a sample from the UK found no improvement in psychological functioning following puberty-suppressing treatment (Biggs, 2020; Carmichael et al., 2021). In this respect, it must be asked whether, given the growing diversity of developmental trajectories, a protocol-based treatment approach can still meet the requirements of more heterogeneous samples.

### Onset Age

EO and LO (Blanchard, 1985; Lawrence, 2003, 2010) are descriptions of the time of the initial manifestation, and the onset age of the first presentation of GD. EO manifests in childhood, before puberty, and is often accompanied (based on clinical experience) by an early gender role change. LO, on the other hand, manifests itself later, after the onset of puberty, and can be more challenging to diagnose from a clinical perspective, as much of the psychosexual development has occurred without recognizable signs of clinical distress. In addition to meeting diagnostic criteria, it is important to consider how the GD developed following a previous acceptance of one's body and how it may interact with coexisting mental health conditions.

The onset age of GD has been considered a marker of diagnostic certainty in the context of an indication for gender-affirming medical interventions in adolescents, although there are no follow-up studies assessing youth with LO developmental trajectories and their mental health outcomes specifically.

Researchers have recently attempted to quantitatively capture the growing heterogeneity of their clientele and the associated developmental trajectories by distinguishing between the temporal duration of GD and the age group at the initial presentation (Arnoldussen et al., 2023; de Rooy et al., 2024; Sorbara et al., 2020). Two studies from the Netherlands (Arnoldussen et al., 2023; de Rooy et al., 2024) showed that among older adolescents (14 years or older at initial presentation), there was an overrepresentation of individuals assigned female at birth (AFAB), greater body-related dissatisfaction, and more psychological problems (measured with the Child Behavior Checklist (CBCL) and Youth Self-Report (YSR), Achenbach, 1991), whereas, within the younger group (younger than 14 years at initial presentation), youth were more likely to have early signs of gender-nonconforming behavior during childhood and more likely to ultimately receive an indication for gender-affirming medical interventions.

It should be noted that Arnoldussen et al. (2023) and de Rooy et al. (2024) also included children before puberty (under the age of 11; as young as 8.9 years) in their studies. Hence, the differences between the younger-presenting and older-presenting groups may arise because children may seek gender identity services primarily because of gender non-conformity, whereas adolescents may be more likely to seek such services because of puberty-related body changes and associated distress. Sorbara et al. (2020) compared two younger and older than 15-year-old age groups and found that the older group of adolescents at initial presentation reported significantly more internalizing psychological problems such as depressive disorders, self-harm, and suicidality and that adolescents presenting at a younger age were significantly more likely to notice their gender incongruence earlier. Sorbara et al. (2020) found no sex differences but AFAB individuals accounted for at least 75% of both groups. Without explicitly capturing onset age as a variable in these studies, the results suggest that older-presenting adolescents are more likely to be AFAB and have psychological problems than younger-presenting adolescents.

While the onset age of GD experiences is a dimension along the timeline, the conceptualization of ROGD (Littman, 2018) has been controversial due to its etiological assumptions. Littman (2018) defined ROGD as a subtype of GD and suggested that GD-related distress resulted less from a persistent and profound cross-gender identification and more from an expression of different psychological problems. ROGD has been defined as the “sudden” onset of GD, often surprisingly to others, without any prior signs. It has been reported predominantly in AFAB adolescents; accompanied by a high rate of psychological problems, especially internalizing problems such as anxiety and depression, and seems to be associated with a strong and urgent desire for gender-affirming medical interventions. Littmann's study has been criticized because the data were collected exclusively on the basis of external assessments by parents who were critical of their children's transgender identity.

Dolotina et al. (2022) and Turban et al. (2023), who attempted to test Littman’s assumptions, came to different conclusions. They asked adults retrospectively about the age of realization of their transgender identity and distinguished between early realization (under 10 years of age) and late realization (10 years and older). In contrast to Littman's study, Dolotina et al. found that the two groups did not differ in most mental health measures, with the group with late realization even reporting less past-year suicidal ideation than the group with early realization of their transgender identity. However, the study’s methods were criticized by Sapir et al. (2024), highlighting the use of incorrect age cohorts and an improper definition of the age of realization. Similar to the findings of Dolotina et al. (2022), a small study from Portugal reported no significant differences in the frequency of co-occurring psychiatric diagnoses between adolescents post- and prepubertal cross-gender behavior (Pereira-Antunes et al., 2023).

### Current Clinical Impressions and Developmental Trajectories

In addition to the “classic” EO developmental trajectories, possibly with a social role change in childhood, less body-related distress until expected puberty, and a relatively higher proportion of individuals assigned male at birth (AMAB), in clinical settings, there seems to be a shifted sex ratio in adolescence in many centers around the world, including our outpatient clinic (84% AFAB vs. 16% AMAB presentations; Hartig et al., 2022; Herrmann et al., 2022; Levitan et al., 2019), with some additional clinical observations from our experience:^1^

1. A considerable number of older adolescents report severe and persistent distress about their sex characteristics with previously gender-conforming psychosexual development and puberty without body-related distress. This group often presents with etiologically unclear psychological problems, such as social fears, depressive withdrawal, and self-injurious behaviors. Inpatient psychiatric treatments and periods of crisis are not uncommon in these cases. While the psychological distress in these cases is notable and should always be taken seriously, multiple factors beyond GD likely contribute to these challenges.
2. In addition, we observe that in a subgroup of AFAB adolescents, one specific body feature (most commonly the breasts) becomes the primary focus for desired change. The self-definition as trans-male is often intellectually derived from a deep feeling of not being able to be female.
3. A third group we encounter, consists of young adolescents in early puberty who, alongside a cross-gender or nonbinary identification, experience intense anxiety about the demands and tasks of the adolescent developmental phase. Their GD often emerges in response to a feeling that they are “not yet able” to meet these demands. In this group, adolescents often have a limited social life, show only little interest in engaging with peers, and demonstrate noticeable inhibition in other areas of their identity.

### Internalizing Psychological Problems

Studies from different countries suggest that more than half of the children and adolescents with a GD diagnosis also have at least one other psychiatric diagnosis (Becker et al., 2014; Chen et al., 2016; de Vries et al., 2011; Holt et al., 2016; Kaltiala-Heino et al., 2015; Nahata et al., 2017; Spack et al., 2012; Wallien et al., 2007). For example, Kaltiala-Heino et al. (2015) reported at least one preexisting or current mental health disorder in 75% of the youth in their study. In German studies, mostly internalizing problems, such as affective and anxiety disorders, self-injury, and suicidality, are also significantly overrepresented compared to norm populations (Becker et al., 2014; Hartig et al., 2022; Levitan et al., 2019; Sievert et al., 2021), both in childhood and adolescence.

Questionnaire assessments, mostly conducted with the CBCL or the YSR (Achenbach, 1991), consistently show levels of psychological problems in the clinical range, regardless of the birth-assigned sex (Bechard et al., 2017; de Graaf et al., 2018; de Vries et al., 2016; Levitan et al., 2019; Zucker et al., 2012). For example, de Graaf et al. (2018, 2022) found consistently elevated rates of psychological problems and suicidality (reported with the CBCL and YSR) among transgender adolescents from the Netherlands, Belgium, Canada, Switzerland, and the UK. Across different countries, there was a clear predominance of internalizing over externalizing problems (de Graaf et al., 2018, 2022; de Vries et al., 2016; Levitan et al., 2019; Röder et al., 2018; Sievert et al., 2021), from which it can be hypothesized that adolescents with GD have higher levels of anxiety, depressive, and somatic complaints as well as suicidality, with fewer aggressive-impulsive problems from the externalizing spectrum in comparison.

Furthermore, there is evidence of the importance of peer relations and family support as key protective factors for psychological well-being. Negative experiences with peers emerged as the most important predictor for psychological problems (Aitken et al., 2016; de Graaf et al., 2018; de Vries et al., 2016; Levitan et al., 2019; Shiffman et al., 2016; Sievert et al., 2021; Steensma et al., 2014), assuming that poor peer relations (PPR) may also be an expression of increased psychosocial problems in general and, as a consequence, increased mental health problems. Although cause and effect are not yet sufficiently understood, adolescents with GD have been identified as being particularly vulnerable to experiences of discrimination and rejection by peers (Toomey et al., 2010) and within the family (Grossman & D’Augelli, 2007).

In addition to age of onset, various attempts have been made to classify GD subgroups based on sexual orientation (Blanchard et al., 1987; Lawrence, 2003, 2010; Nieder et al., 2011). This approach was predicated on the supposition that the original "true transsexualism" (Benjamin, 1966) was concomitant with an “opposite-sex”/heterosexual orientation (having undergone gender-confirming medical interventions). Nieder et al. (2011) found that in AFAB adults with EO GD, more than 90% reported a gynecophilic orientation, compared to only 50% in LO GD. In contrast, AMAB adults showed a more ambiguous picture with a similar proportion of gynecophilic and androphilic orientation in EO and a predominantly gynecophilic orientation in LO.

From a developmental psychology perspective, clarity about one's sexual attraction is an important step in adolescent maturation. This process requires young people to be able to develop relative freedom, both interpersonally and intrapersonally (Erikson, 1968). It is about coming to terms with oneself, one's own body, and one's sexual desires toward others. In this sense, clarity about sexual orientation can also be seen as a sign of the consolidation of adolescent identity.

### Summary and Derivation of the Research Questions

Previous research suggests that the differentiation of subgroups or clinical clusters of adolescents with GD may improve our understanding of heterogeneous developmental pathways and enable more individualized care and treatment steps. This seems particularly necessary given the increasingly variable clinical manifestations and resulting different needs that find little place in current treatment protocols. Therefore, the present study aims to systematically assess a large sample of adolescents with GD concerning their clinical characteristics of onset age and internalizing problems in the German-speaking area. The studies will investigate the relationship between self-reported (and then later clinically rated) onset age and internalizing problems in adolescents, while controlling for other possible influencing factors, such as birth-assigned sex, age at assessment, intensity of GD, body satisfaction, PPR, family functioning level, and sexual orientation. Based on previous research and clinical impressions, we hypothesize that a LO (as opposed to an EO) would be related to more internalizing problems.

The following three research questions will be answered in this article:

1. What is the percentage of different onset age (EO vs. LO) among adolescents with GD presenting to a specialized gender identity service?
2. What is the percentage of clinically relevant internalizing problems in adolescents with GD (compared to the norm population)?
3. What is the relationship between onset age and internalizing problems in adolescents with GD, while controlling for other possible influencing factors, such as birth-assigned sex, age at assessment, intensity of GD, body satisfaction, poor peer relations, family functioning level, and sexual orientation?

In addition, exploratory analyses examined the association between age of onset and externalizing problems, total problems, and clinician-reported global functioning, and investigated different criteria for a LO.

## Method

### Participants

**Fig. 1:**
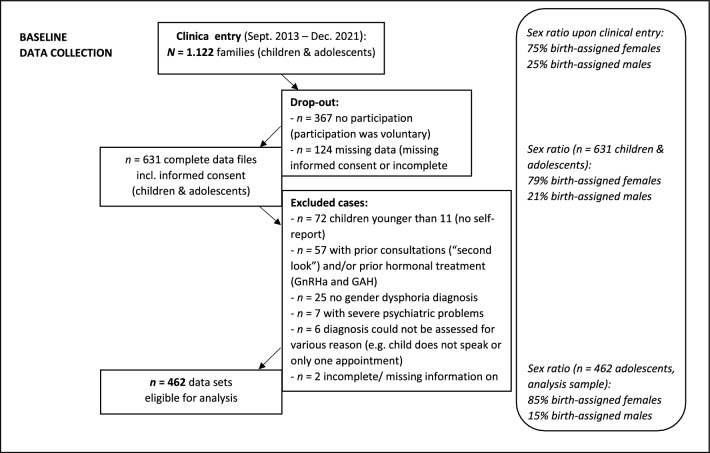
Participants and sex ratios at the Hamburg GIS for children and adolescents

The current study examined adolescents who presented to the Hamburg Gender Identity Service for Children and Adolescents (Hamburg GIS) between September 2013 and December 2021, participated in the study, and met diagnostic criteria for GD according to the DSM-5 (APA, 2013). GD diagnoses were rated by clinical experts using standardized diagnostic checklists after a diagnostic period of several months. The referral population comprised a total of 1122 children (aged 5 to 10 years) and adolescents (aged 11 years and above) during the survey period. A total of 631 datasets were collected from these families. There were no significant age (*t*(557) = −1.12, *p* = 0.265, *d* = −0.13) or sex differences (*χ*^2^ (1, *N* = 559) = 1.77, *p* = 0.183, *OR* = 0.69) between included and excluded adolescents. For various reasons, 169 cases had to be excluded (Fig. 1). Thus, the present study included the complete data of 462 treatment naïve adolescents aged 11–18 years with a GD diagnosis and their families. The median year of assessment was 2018.

### Procedure

Since 2013, all families presenting to the Hamburg GIS have been invited to participate in our study. Participation is voluntary, and data collection takes place during the first appointment, before the start of a diagnostic phase, and any gender-affirming medical intervention. The study follows a cross-sectional design with the use of internationally established, psychometrically tested (self-report) questionnaires alongside clinicians’ ratings.

### Measures

#### Sociodemographics and Onset Age

Sociodemographic characteristics were: birth-assigned sex, age at first presentation, nationality, parents’ marital status, living situation, and socioeconomic status (SES) assessed with the Winkler Index (Winkler & Stolzenberg, 1999; for a more detailed description, see Herrmann et al., 2023a, b; Levitan et al., 2019).

In addition, cross-gender identification (experiences of belonging to the gender that is “opposite” to the birth-assigned sex) was recorded for all participants using a sum score, as it has been done in previous studies (e.g., de Vries et al., 2016): in the YSR, Items 5 and 110 refer to cross-gender identification (“I act like the opposite sex” and “I wish I were of the opposite sex”). The sum score of the two items can be used as a measure of the intensity of cross-gender identification. The score ranges from 0 to 4, with larger values expressing a higher intensity of gender incongruence (given a binary understanding of gender identity).

To obtain information on onset age, specialized clinicians assessed the criteria for GD in childhood and adolescence according to the DSM-5 with the help of a study-specific checklist during the diagnostic sessions with the adolescents, in which they described their own gender identity and GD development. Later, onset age was operationalized by dichotomous assignment to EO if the DSM-5 diagnosis of GD was already applicable in childhood/before puberty (defined for this purpose as before the age of 12 years) or to LO in participants who retrospectively did not meet the criteria for GD in childhood but did in adolescence after the onset of puberty (defined for this purpose as age of 12 years and older). Thus, two groups of participants could be classified for the study: adolescents with an EO developmental trajectory (who met DSM-5 criteria of GD in both childhood and adolescence), and adolescents with a LO (who met DSM-5 criteria of GD only in adolescence). In additional exploratory analyses, we later subdivided the LO group by GD duration (i.e., for how long the individuals had fulfilled the various GD criteria in adolescence) and labeled all participants whose GD had been present for less than one year as “recent onset.” For the classification of recent onset cases, we only used the first four A criteria of the GD diagnosis to focus on the incongruence between gender identity and body characteristics (and not the gender role).

#### Internalizing Problems

Internalizing problems were assessed using the 1991 German version of the YSR (Achenbach, 1991; Döpfner et al., 1998) for adolescents aged 11–18 years. The YSR consists of 120 items rated on a 3-point scale ranging from 0 = “not true” to 2 = “very true or often true.” The total sum of all problems is reflected in the total problem score, which can be divided into two main scales (internalizing and externalizing problems). In addition, values in the clinical range (> 90th percentile; *T* scores > 63) can be given for these three indices. Norm values of adolescents (aged 11–18 years) from the German general population are available for the different indices and birth-assigned sex (Döpfner et al., 1998). These norm or *T* values can be used to determine whether the values of our sample are within the normal range. Cronbach’s α for the internalizing scale was 0.91 in the present sample. For exploratory purposes, the YSR score for externalizing problems and the total problem score were also calculated to examine psychological functioning/problems more broadly. In this study, Cronbach’s α for the externalizing and total problem scales was 0.83 and 0.93, respectively.

The Children’s Global Assessment Scale (CGAS) (Shaffer et al., 1983) was used within the exploratory analyses to assess adolescent global functioning using clinicians’ ratings. The CGAS is one of the most widely used assessment scales for measuring everyday functioning in children and adolescents (Schorre et al., 2004). Clinicians are asked to score the patient's most impaired level of general functioning over the past four weeks on a health-illness continuum. The instrument is divided into 10-point intervals ranging from 10 (“needs constant supervision”) to 100 (“superior functioning in all areas”), with higher scores (above 80) indicating good global functioning.

#### Control Variables

Three items from the YSR were used to assess poor peer relations (PPR): Item 25 (“I don’t get along with other kids”), Item 38 (“I get teased a lot”), and Item 48 (“I am not liked by other kids”). The PPR was developed by Zucker et al. (1997) and has been used in previous studies to measure PPR in youth with GD (de Vries et al., 2016; Levitan et al., 2019; Sievert et al., 2021; Zucker et al., 2002, 2012). The index ranges from 0 to 6, with higher scores reflecting poorer peer relations. In the present study, Cronbach’s *α* was 0.66.

For general family functioning (GFF), the McMasters’ Family Assessment Device (FAD) (Epstein et al., 1983) was used. Only the GFF subscale was evaluated. The GFF scale comprises 12 items, such as family acceptance (“Individuals are accepted for what they are”), rated on a 4-point scale (from 1 = “strongly agree” to 4 = “strongly disagree”). The sum of the 12 items was divided by 12 to create a score ranging from 1 to 4, with higher scores indicating lower levels of family functioning. The cutoff for categorical analyses (problematic or unhealthy family functioning) is 2.17 (Byles et al., 1988). Cronbach’s *α* was 0.88 in the present sample.

The pictorial measure Hamburg Body Drawing Scale (HBDS) was used to assess body satisfaction (Appelt & Strauß, 1988; Becker et al., 2016). Participants were asked to rate their satisfaction with various body features and overall appearance on a 5-point scale (from 1 = “very dissatisfied” to 5 = “very satisfied”). The HBDS has been revised and validated for transgender populations (Becker et al., 2016). Internal consistency for the HBDS subscales (Cronbach’s α = 0.63–0.91) is satisfactory (Becker et al., 2016). In the present study, only one item on satisfaction with the overall appearance (“satisfaction with the overall appearance”) was used.

Sexual orientation was assessed with a self-developed item asking about physical (sexual) attraction in partner choice (“To whom are you more physically [sexually] attracted?”) and providing six response categories: “to no one,” “to girls,” “more to girls, sometimes to boys,” “to both girls and boys,” “to boys,” and “other” (free text response). From these, different categories of sexual orientation were formed in relation to birth-assigned sex: same-sex, opposite-sex, bisexual, asexual or unsure, and pansexual. Later, two categories were formed for the regression analysis: “same-sex” vs. “other” (based on birth-assigned sex).

### Statistical Analysis

Confidence intervals (95% CIs) were calculated for the percentage of EO and LO. For continuous variables, two-way analyses of variance were calculated to explore differences in the sociodemographic and psychosocial characteristics between birth-assigned sex (AFAB, AMAB) and onset age groups (EO, LO). Exploratory chi-square tests were conducted for categorical variables, with Fisher–Freeman–Halton exact tests used when subsample sizes were too small. Standardized effect sizes (partial eta squared [η_p_^2^], odds ratios [ORs], and Cramér’s *V*) were calculated to quantify the magnitude of the effect.

Internalizing problems were assessed using raw scores, *T* scores, and clinical ranges (> 90th percentile; *T* scores > 63). In addition, confidence intervals for *T* scores were calculated to compare the present sample with the age and sex equivalent German norms (Döpfner et al., 1998). Whenever the 95% CIs were not within the normal range of the *T*-distribution (*M* = 50, *SD* = 10), a significant difference from the reference group can be assumed. When the 95% CIs overlapped, the results were not significantly different from each other (Cumming & Finch, 2005). Against the background of the literature, the study focused on internalizing problems. However, we additionally evaluated the externalizing scale and total problem score as presented for exploratory purposes.

A multiple linear regression analysis was performed to examine our hypothesis, i.e., the predictive value of onset age for internalizing problems. For this purpose, the raw scores of the YSR internalizing scale were used, and we controlled for birth-assigned sex, age, PPR, GFF, sexual orientation, body satisfaction, and cross-gender identification. In the exploratory analyses, the same control factors and tests were used to examine the associations of onset age with externalizing problems and the total problem score. For the total problem score, three items on PPR (Items 25, 38, and 48) were excluded because PPR was a separate predictor in the model. An a priori power analysis (using G*Power) showed that a small effect (*f* = 0.02) with a power of 85% could be tested in a multiple regression analysis with 462 cases and eight predictors.

In the exploratory analyses, a multiple linear regression analysis was also conducted to examine the association between onset age (independent variable) and global functioning (dependent variable). The control variables described above were used.

Assumptions for the statistical analyses were checked and met. Individual missing values were replaced using the expectation–maximization algorithm (Little & Rubin, 2014). All statistical analyses were performed using SPSS version 27.

## Results

### Description of the Sample and Onset Age

Table 1 shows the details of the sociodemographic and clinical characteristics of the participants. The sample (*n* = 462) consisted of 85% AFAB and 15% AMAB adolescents with a mean age of 15.5 years. EO was present in 51% of participants and LO in 49%. In our study, AMAB (but not AFAB) adolescents with an LO were significantly older at initial presentation than those with an EO. There was no significant association between birth-assigned sex and onset age (*χ*^2^(1, *N* = 462) = 0.25, *p* = 0.620, *OR* = 0.88).

**Table 1:** Sociodemographic and psychosocial characteristics as a function of birth-assigned sex (assigned female vs. male) and onset age (early vs. late onset)

	AMAB (*n* = 70)			AFAB (*n* = 392)			Combined (*n* = 462)			Comparison by sex				Comparison by onset age				Interaction (sex x onset age)			
	*M*	*SD*	*n*	*M*	*SD*	*n*	*M*	*SD*	*n*	*F*	*df*	*p*	η_p_^2^	*F*	*df*	*p*	η_p_^2^	*F*	*df*	*p*	η_p_^2^
*Age at assessment (in years)*																					
Early onset	14.92	1.71	34	15.23	1.54	203	15.18	1.56	237												
Late onset	16.33	1.15	36	15.64	1.33	189	15.75	1.32	225												
Combined	15.65	1.61	70	15.42	1.45	392	15.46	1.48	462	1.08	1, 458	.299	.00	23.88	1, 458	< .001	*.*05	7.30	1, 458	.007	.02
*Parental socioeconomic status (Winkler Index)*																					
Early onset	6.21	1.65	34	6.25	1.59	203	6.24	1.60	237												
Late onset	6.94	1.57	36	6.81	1.59	189	6.83	1.58	225												
Combined	6.59	1.64	70	6.52	1.61	392	6.53	1.61	462	0.05	1, 458	.829	.00	9.85	1, 458	.002	.02	0.19	1, 458	.663	.00
*Poor peer relations (YSR)*																					
Early onset	2.26	1.52	34	1.27	1.47	203	1.41	1.52	237												
Late onset	1.94	1.49	36	1.73	1.39	189	1.76	1.40	225												
Combined	2.10	1.51	70	1.49	1.45	392	1.58	1.47	462	10.40	1, 458	.001	.02	0.14	1, 458	.711	.00	4.33	1, 458	.038	.01
*General family functioning (FAD)*																					
Early onset	1.90	0.59	34	1.87	0.61	203	1.88	0.61	237												
Late onset	1.99	0.46	36	2.03	0.57	189	2.02	0.55	225												
Combined	1.95	0.52	70	1.95	0.60	392	1.95	0.59	462	0.00	1, 458	.992	.00	2.58	1, 458	.109	.01	0.18	1, 458	.671	.00
*Body satisfaction (HBDS)*																					
Early onset	2.62	0.91	34	2.37	0.84	203	2.41	0.85	237												
Late onset	2.15	0.57	36	2.45	0.80	189	2.40	0.77	225												
Combined	2.38	0.79	70	2.41	0.82	392	2.41	0.81	462	0.05	1, 458	.824	.00	3.53	1, 458	.061	.01	6.75	1, 458	.010	.02
*Cross-gender identification (YSR)*																					
Early onset	3.76	0.50	34	3.90	0.33	203	3.88	0.36	237												
Late onset	3.53	0.65	36	3.75	0.60	189	3.71	0.61	225												
Combined	3.64	0.59	70	3.83	0.49	392	3.80	0.51	462	7.58	1, 458	.006	.02	9.26	1, 458	.002	.02	0.40	1, 458	.528	.00

The Winkler Index ranges from 3 to 9 (9 = highest socioeconomic status), the poor peer relations score from 0 to 6 (6 = worst peer relations), the FAD from 1 to 4 (4 = lowest levels of family functioning), the HBDS from 1 to 5 (5 = highest level of body satisfaction), and the cross-gender identification sum from 0 to 4 (4 = highest level of cross-gender identification)

*AFAB/AMAB* assigned female/male at birth, *FAD* McMaster Family Assessment Device, *HBDS* Hamburg Body Drawing Scale, *YSR* Youth Self-Report

Almost all adolescents were German citizens (96%) and came from a family with a medium (57%) or high SES (30%). Participants with a LO had a significantly higher parental SES than participants with an EO. When asked about peer-related problems (PPR) in the past six months, AFAB adolescents in the LO group reported significantly more problems than those in the EO group, whereas no such difference was observed among AMAB adolescents, as indicated by a significant interaction effect. The GFF subscale showed that family interactions/functioning were, on average, unproblematic (scores below the cutoff at 2.17 for all groups), with no significant group differences. Body satisfaction was, on average, low, regardless of the birth-assigned sex or onset age, but there was a significant interaction effect. AMAB adolescents in the EO group reported higher body satisfaction than those in the LO group, while there was no significant difference between the AFAB EO and LO groups. Groups differed in cross-gender identification or the intensity of gender incongruence, respectively: it was significantly more pronounced in AFAB adolescents than in AMAB adolescents and among EO adolescents compared to LO adolescents.

In terms of SO, half of the participants reported a same-sex sexual orientation (in relation to birth-assigned sex) and about a quarter reported an opposite-sex sexual orientation. The rest reported a bisexual or pansexual sexual orientation or were asexual or unsure. There were significant differences between the onset age groups in the present study: Significantly more EO youth (63%) were same-sex oriented than LO youth (30%); the latter were typically opposite-sex oriented (35%) or bisexual (18%). There were no significant sex differences. Tables 4 and 5 show more information on sexual orientation.

### Internalizing Problems

Results for internalizing problems are shown in Table 2. In reference to the German norm population (*M* = 50, *SD* = 10), adolescents with GD had significantly higher *T* scores (95% CI without *M* = 50) for internalizing problems. Adolescents with GD scored, on average, more than 1.5 *SD* higher on the internalizing problems scale than peers from the YSR reference group.

**Table 2:** Internalizing problems (YSR) as a function of birth-assigned sex and onset age and compared to German norm scores

	Raw scores				*T* scores (adolescents with GD with reference to the norm)				Clinical range (*T* scores > 63)	
	*M*	*SD*	*n*	95% CI	*M*	*SD*	*n*	95% CI	%	*n*
*AMAB*										
Early onset	19.24	8.63	34	[16.22; 22.25]	67.12	9.61	34	[63.76; 70.47]	70.6	24
Late onset	22.92	8.69	36	[19.98; 25.86]	71.22	9.45	36	[68.03; 74.42]	80.6	29
Combined	21.13	8.80	70	[19.03; 23.23]	69.23	9.68	70	[66.92; 71.54]	75.7	53
*AFAB*										
Early onset	19.41	10.71	203	[17.93; 20.89]	63.12	10.82	203	[61.62; 64.62]	45.3	92
Late onset	25.12	11.35	189	[23.49; 26.74]	69.30	11.28	189	[67.68; 70.92]	64.0	121
Combined	22.16	11.37	392	[21.03; 23.29]	66.10	11.45	392	[64.96; 67.24]	54.3	213
*Combined*										
Early onset	19.38	10.42	237	[18.05; 20.72]	63.69	10.73	237	[62.32; 65.06]	48.9	116
Late onset	24.76	10.98	225	[23.32; 26.21]	69.61	11.01	225	[68.16; 71.06]	66.7	150
Combined	22.00	11.02	462	[21.00; 23.01]	66.57	11.25	462	[65.55; 67.60]	57.6	266

Age and birth-assigned sex equivalent German norm YSR *T* scores with *M* = 50 and *SD* = 10 were derived from Döpfner et al. (1998). Raw scores for the internalizing scale range from 0 to 62, and *T* scores range from 25 to 100. For the clinical range, the percentage and total number of individuals scoring within the clinical range of internalizing problems are presented. These individuals scored lower than 89% of the age and birth-assigned sex equivalent reference group

*AFAB/AMAB* assigned female/male at birth, *GD* gender dysphoria, *YSR* Youth Self-Report

Regarding onset age, LO adolescents reported significantly more internalizing problems than EO adolescents. A closer examination of the 95% CIs indicated that these differences were only present in AFAB adolescents but not in AMAB adolescents (nonoverlapping 95% CIs). In the LO group, 67% scored within the clinical range, whereas only 49% of the EO group did (*T* scores > 63). Overall, 58% of adolescents in the sample had clinically relevant levels of internalizing problems. There were no significant sex differences (overlapping 95% CIs).

### Association between Onset Age and Internalizing Problems

The results of the multiple linear regression analysis used to test our hypothesis are shown in Table 3. The regression analysis revealed a significant association between onset age and internalizing problems. As hypothesized, a LO (as opposed to an EO) was associated with reporting significantly more internalizing problems: having a LO was related to scoring, on average, three points higher on the internalizing scale of the YSR (YSR raw scores). Concerning the control variables, more internalizing problems were significantly related to birth-assigned sex (AFAB), PPR (higher degrees of poor peer relations), GFF (lower family functioning levels), body satisfaction (lower degrees of body satisfaction), and sexual orientation (no same-sex sexual orientation). Overall, the model explained 44.5% of the variance in internalizing problems: onset age explained 1.5% of the variance, which can be interpreted as a small effect, and the control variables explained a total of 43%.

**Table 3:** Association between internalizing problems (YSR raw scores) and the onset age

	*b*	*SE b*	95% CI for* b*	*ß*	*p*
Intercept	6.54	5.88	[− 5.02; 18.10]		.267
Birth-assigned sex (0 = male, 1 = female)	2.69*	1.09	[0.55; 4.83]	.09	.014
Age in years	− 0.06	0.27	[− 0.60; 0.47]	− .01	.819
Poor peer relations (YSR)	2.67***	0.28	[2.11; 3.22]	.36	< .001
General family functioning (FAD)	5.96***	0.71	[4.57; 7.35]	.32	< .001
Body satisfaction (HBDS)	− 2.68***	0.51	[− 3.67; − 1.68]	− .20	< .001
Cross-gender identification (YSR)	0.56	0.78	[− 0.98; 2.10]	.03	.477
Sexual orientation (0 = same-sex, 1 = other)	2.22**	0.82	[0.61; 3.83]	.10	.007
Onset age (0 = early, 1 = late)	3.00***	0.84	[1.35; 4.65]	.14	< .001

*p < .05, **p < .01, ***p < .001*, FAD* McMasters’ Family Assessment Device, *GD* gender dysphoria, *HBDS* Hamburg Body Drawing Scale, *YSR* Youth Self-Report

### Exploratory Data Analyses: Onset Age, Externalizing Problems, Total Problems Score, and Global Functioning

In exploratory analyses, the duration of the experienced GD was examined only within the group of LO adolescents. Between 23 and 36% of the adolescents met the criteria for a recent onset, which implies that the onset of GD had occurred less than 1 year ago (based on clinicians’ interviews with adolescents; see Table 4, 5 and 6).

Additional exploratory data analyses were conducted to examine the associations between onset age and externalizing problems, as well as the total problem score and global functioning (CGAS) (Appendix Tables 7–12). Externalizing problems were less common than internalizing problems in adolescents with GD but were still elevated compared with the norm population (Table 7). Clinically relevant externalizing problems were reported by 15% of participants. There were no significant differences between EO and LO youth but AFAB adolescents scored significantly higher on the externalizing scale than AMAB adolescents. Also elevated was the total problem score: adolescents with GD scored more than 1 *SD* higher than the reference group and 46% of participants were in the clinical range. LO adolescents reported, on average, significantly higher total problem scores and tended to be more often in the clinical range than EO adolescents. The differences were particularly evident in AFAB adolescents. There was also a significant difference in global functioning between the two onset age groups in AFAB adolescents (but not in AMAB adolescents), with AFAB adolescents in the LO group having a significantly lower level of global functioning than AFAB adolescents in the EO group (Table 8).

In multiple regression analysis for externalizing problems, a significant association was identified between onset age and externalizing problems (Table 9). An inverse association was found with internalizing problems, that is, LO adolescents reported significantly fewer externalizing problems. Significant control variables were birth-assigned sex, PPR, and GFF. The model explained 16% of the variance in externalizing problems: onset age explained 1% of the variance (small effect) and the control variables explained 15%.

Another regression model (Table 10) tested whether onset age affected the total problem score and global functioning level. Onset age and total problem score were not associated. Significant control variables were birth-assigned sex, PPR, GFF, and body satisfaction. The model explained 39% of the variance in the total problem score.

Table 11 shows the results for the association between onset age and global functioning. LO proved to be a significant predictor of a worse global functioning as rated by clinicians. Significant control variables were PPR and cross-gender identification/intensity of gender incongruence. The model explained 11.6% of the variance in the CGAS: onset age explained 2.2% (small effect) and the control variables explained 9.4%.

Last, we explored whether onset age was related to internalizing problems when the recent onset and LO groups were considered separately (Table 12). Belonging to the LO group (as opposed to the EO group) was associated with significantly more internalizing problems. Belonging to the recent onset group (GD present for less than one year) as opposed to the EO group was not associated with more internalizing problems but showed a tendency to do so. Significant control variables were birth-assigned sex, PPR, GFF, body satisfaction, and sexual orientation. The model explained a total of 44.5% of the variance in internalizing problems, of which onset age accounted for 1.4% (small effect) and the control variables for 43.1%.

## Discussion

The present study aimed to assess the frequency of EO vs. LO developmental trajectories, and to investigate the association between the onset age of GD and internalizing problems in adolescents attending a specialized outpatient clinic for GD. Our findings identified onset age as a significant predictor of psychological problems, with LO in adolescence associated with higher levels of internalizing problems.

The higher levels of internalizing problems in the LO group than in the EO group correspond to recent findings in which a group classification was made based on the age of presentation (Arnoldussen et al., 2023; de Rooy et al., 2024; Sorbara et al., 2020). In these studies, older-presenting youth were more likely to have psychological problems (depressive and anxiety disorders as well as higher CBCL and YSR scores) than the younger-presenting ones (de Rooy et al., 2024; Sorbara et al., 2020), similar to the higher *T* scores and the higher percentage of adolescents scoring in the clinical range of internalizing problems in the LO group (as opposed to the EO group) in our sample. Sorbara et al. (2020) put forward two hypotheses for these differences: First, the older-presenting group was more advanced in pubertal development and, therefore, more distressed than the younger-presenting group. Second, older-presenting youth may experience more minority stress and less family support and hence present at an older age than younger-presenting youth (de Rooy et al., 2024). In contrast, our EO and LO groups did not significantly differ in age (15.2 years to 15.7 years at first presentation). In addition, we found that a longer duration of GD with onset already in childhood (EO) was not associated with reporting more but less internalizing problems.

In line with the present results, a study by Dolotina et al. (2022) found a roughly even distribution of onset age. However, in contrast to our findings, the group with later realization of their gender identity reported significantly less suicidal thoughts than the group with earlier realization, and no significant differences were found between groups for other mental health measures. That said, comparisons between these two studies should be made carefully, considering the differences in methods (e.g., definition of onset age, sample selection), and the critique by Sapir et al. (2024).

Contrary to our expectations, the sex ratio did not differ between the EO and LO groups but was balanced with a share of 86% and 84% AFAB, respectively. In this respect, it cannot be assumed that the LO group shows an overrepresentation of AFAB adolescents. On the other hand, the sex ratio in our study should be considered with limitations: the physical maturation and the fact that girls enter puberty much earlier than boys and usually develop recognizable secondary sexual characteristics well before the age of 12 (Grüters-Kieslich, 2009) points to a methodological problem in the classification of onset age when this is recorded using age cutoffs, as we did. Thus, AFAB adolescents may be overrepresented in the EO group due to the earlier onset of puberty. Notably, the EO group reported a greater intensity of gender incongruence and cross-gender identification with otherwise lower internalizing problems. This could correspond to the assumption of Cohen-Kettenis and Klink (2015) that there is a clinical subtype of AFAB adolescents with an EO course and exceptionally strong GD who have an early desire for gender-affirming medical interventions. At the same time, however, this also shows that GD with high intensity and resulting distress does not necessarily have to be connected to strong internalizing problems but exhibits different or independent developmental pathways under certain circumstances. A clinical approach to understanding seems to be urgently required to more precisely describe these developmental trajectories and to be able to make well-founded treatment decisions on this basis.

Considering the findings of previous studies of onset age, a dynamic developmental link between AFAB and LO was suggested but not shown in our study (i.e., no significant association between birth-assigned sex and onset age). Nieder et al. (2011) reported an EO rate of 78% in an adolescent sample of AFAB individuals presenting to specialized European gender identity clinics. In contrast, our EO proportion of 51% is not only lower but also contrary to the notion that increased societal openness and information about transgender people might result in an earlier age-related reflection on the potential discrepancy between birth-assigned sex and gender identity (e.g., Aitken et al., 2015). Rather, contrary to this expectation, there appears to be a temporal shift to later adolescence, when the incongruence is first perceived (Sun et al., 2023).

However, it is possible that the decrease in stereotypical role expectations and evolved possibilities of expression can also be understood ambiguously: while this is associated in some with relief from inflexible gender role expectations and more individual freedom, it may lead others to a loss of orientation and the search for new identifications to counteract the insecurity. In both cases, the unresolved question arises as to how it is possible to experience puberty without distress in the case of a LO and how it generally leads to the development of dysphoria.

Moreover, transgender adolescents spend a considerable amount of time online and on social media (Herrmann et al., 2023a), where the notion of one right way to be transgender may be reinforced, often through a focus on binary-presenting individuals and medical transitions (Etengoff, 2019). As a result, it may be challenging for adolescents to shape their gender identities and experiences in a fully independent and individual way, separate from the often binary gender expressions and expectations they encounter online.

Exploratory analyses of our study suggest that AFAB adolescents with a LO reported, in addition to high levels of internalizing problems, significantly more peer problems than those with an EO. Identity is a process essentially determined by interactions (Mertens, 1996), both between the individual and relevant others and within the self in the process of reflecting on and testing different conceptions of self. A consolidated and stable experience of identity, also concerning a mature clarity about one’s own gender identity, is accordingly clearly more difficult for individuals with high symptomatic stress and conflictual peer relations. It can be assumed from this that LO adolescents represent a particularly vulnerable group that would benefit from an individually tailored treatment plan, in addition to long-term psychotherapeutic support that will hopefully help them to consolidate their own identity, irrespective of their gender, and create space for development again.

### Clinical Implications

Our results raise three major questions: (1) Is there a clinical LO subtype?; (2) On what basis or under what conditions can reliable indications for gender-affirming medical interventions be made?; and (3) Can a protocol-based approach still meet the needs of individuals seeking treatment given the diversity of clinical presentations and the different developmental pathways that can be derived from them?

First, the present findings indicate the presence of increasing heterogeneity not only in clinical presentations of treatment seekers but also within the sample between EO and LO pathways. In our study, the LO group reported significantly more internalizing psychological and peer problems (at least AFAB adolescents) but less cross-gender identification than the EO group. Other influences are likely since neither the duration of the experience of GD nor the intensity of cross-gender identification can be convincingly assumed to be causal for the overall very high rate of internalizing problems.

Drawing on the clinical manifestations briefly outlined at the beginning, we can assume that for a substantial proportion of adolescents with long psychiatric histories, LO will have a different developmental trajectory than a deeply felt incongruence between body and gender identity. Clinical observations at our center suggest that the proportion of LO (as opposed to EO) adolescents has increased in recent years. Further (longitudinal) studies are needed to identify possible shifts in developmental pathways.

In contrast to Littman (2018), we understand different developmental pathways of GD as etiologically relevant but not necessarily milder in course or less in need of treatment. Rather, the question is: What interventions would benefit this particularly vulnerable group of youth with LO development beyond the desire for medical intervention?

Second, an indication for gender-affirming medical interventions based purely on descriptive and external criteria appears neither purposeful nor feasible given the reciprocal overlap of GD experiences and other developmental conflicts (Edwards-Leeper, 2017; Edwards-Leeper & Smith, 2017; Zucker & Bradley, 1995). Our proposal is therefore a comprehensive, process-oriented diagnostic phase that includes psychosexual development, narratives about one’s identity development, and a detailed anamnesis for other relevant adolescent issues and conflicts. This diagnostic process would form the basis of an assessment of the developmental course, in the context of which considerations about gender-affirming interventions can be discussed. The focus would be on the interaction between GD and relevant developmental conflicts, allowing for a deeper understanding of the individual's journey and the development of an individual narrative. Co-occurring psychological problems do not represent a clear contraindication for gender-affirming medical interventions, as long as they can be understood in terms of developmental dynamics. Nonetheless, the psychodynamic perspective and clinical experience suggest that a substantial proportion of adolescents at the time of the diagnostic phase may benefit less from physical interventions than from adjunctive and developmental psychotherapy—considering how many cases present co-occurring psychological problems. Given concerns about increasing numbers of detransitions (Cohn, 2023), persistent distress or regret despite gender-affirming medical interventions (Roberts et al., 2022), and ambiguity regarding the stability of the GD experience over time, cautious and intensely reflective indications for this group of adolescents appear essential.

Third, although our total sample had similarly high levels of internalizing problems as most samples from other European gender identity services (see de Graaf et al., 2018), the group of LO adolescents is characterized by particularly elevated levels of internalizing problems (67% scoring in the clinical range vs. 57% of the total sample). Abbruzzese et al. (2023) question the applicability of the Dutch results to nowadays more heterogeneous, non-preselected treatment groups, which include youth with a LO developmental trajectory and severe psychological problems. The growing diversity of developmental trajectories in adolescents, with varying intensity of gender dysphoric distress and co-occurring, with GD interacting psychological problems, demonstrates the need for an individualized treatment setting. The guiding question “What works for whom, at what time, and in what setting” enables a differential indication (Dorr et al., 2020) for outpatient or inpatient psychotherapy, gender-affirming medical interventions, or low-frequency counseling/support oriented to individual needs.

### Limitations

Our findings should be considered in light of several limitations. The cross-sectional design of our study cannot provide information on long-term trajectories or influencing factors. This is especially true for the control variables studied, such as GD intensity, sexual orientation, and body satisfaction, which should be considered snapshots and may change over time.

Another difficulty lies in the operationalization of LO and EO trajectories. Unfortunately, the literature lacks a clear conceptualization/definition and measurement of onset age in adolescents. Therefore, onset age was retrospectively assessed by evaluating the clinicians’ ratings on the DSM-5 criteria for childhood after conducting a comprehensive diagnostic process with the adolescents, in which they described their gender identity development. However, the cutoff age of 12 years for EO vs. LO was artificially set. Given the age difference in puberty onset in AFAB and AMAB adolescents and the now advanced timing of AFAB pubertal development, the cutoff age is one of our limitations. For future studies, a clearer determination of puberty based on the development of physical/sex characteristics would be useful.

Similarly, while the collection of internalizing problems using YSR data is widespread, a categorical diagnostic using standardized questionnaires would be important. Another aspect that should be considered in future studies is the systematic collection of data on the course of treatment since this will make it clearer to what extent there are also differences in the indication of treatment and treatment trajectories.

Data interpretation is also limited by the high and increasing proportion of AFAB youth, accounting for 85% of the present study/analysis sample (see Fig. 1). As a result, the subsample of AMAB adolescents in the present study was relatively small. Furthermore, the sample sizes for our hypothesis-testing analysis (multiple linear regression) were, in some instances, unbalanced (e.g., AFAB vs. AMAB) but still sufficient (more than 10%). However, the descriptive and exploratory analyses (e.g., evaluating the 95% CIs of the YSR scores) should be interpreted cautiously (estimates are less reliable in small samples, such as AMAB adolescents with LO).

### Conclusions

The present study showed that about half of the adolescents attending a specialized gender identity service reported an LO course. Adolescents with a GD diagnosis reported, on average, significantly more internalizing problems than adolescents from the norm population, and their scores often fell within the clinical range. Furthermore, a LO developmental trajectory was associated with particularly high levels of internalizing problems.

Our findings indicate a specific subgroup of LO adolescents in the growing spectrum of heterogeneous developmental trajectories. The diverse trajectories and the presence of particularly vulnerable subgroups highlight the need to move away from a single protocol-based approach toward a more individualized approach to indications that are based on developmental dynamics during adolescence.

## Appendix

See Tables 4, 5, 6, 7, 8, 9, 10, 11, and 12.

**Table 4:** Sexual orientation as a function of birth-assigned sex (assigned female vs. assigned male) and onset age (early vs. late onset).

	Early onset		Late onset		Combined		Group comparisons		
	(*n* = 237)		(*n* = 225)		(*n* = 462)		Test	*p*	Effect
Sexual orientation (in relation to birth-assigned sex)	%	*n*	%	*n*	%	*n*			
Same-sex	63.3	150	29.8	67	47.0	217			
Opposite sex	11.8	28	34.7	78	22.9	106			
Bisexual	8.0	19	18.2	41	13.0	60			
Asexual or unsure	15.2	36	13.8	31	14.5	67			
Pansexual	1.7	4	3.6	8	2.6	12	*χ*^2^(4, 462) = 64.84	<.001	*V* = .38***

	AMAB		AFAB		Combined		Group comparisons		
	(*n* = 70)		(*n* = 392)		(*n* = 462)		Test	*p*	Effect
Sexual orientation (in relation to birth-assigned sex)	%	*n*	%	*n*	%	*n*			
Same-sex	48.6	34	46.7	183	47.0	217			
Opposite sex	22.9	16	23.0	90	22.9	106			
Bisexual	7.1	5	14.0	55	13.0	60			
Asexual or unsure	20.0	14	13.5	53	14.5	67			
Pansexual	1.4	1	2.8	11	2.6	12	*χ*^2^(4, 462) = 4.37	.358	*V* = .10

****p* < .001, AFAB/AMAB = assigned female/male at birth.

**Table 5:** Sexual orientation as a function of onset age (early vs. late onset) for adolescents assigned male vs. female at birth

Assigned male at birth adolescents	Early onset		Late onset		Combined		Group comparisons		
	(*n* = 34)		(*n* = 36)		(*n* = 70)		Test	*p*	Effect
Sexual orientation (in relation to birth-assigned sex)	%	*n*	%	*n*	%	*n*			
Same-sex	58.8	20	38.9	14	48.6	34			
Opposite sex	14.7	5	30.6	11	22.9	16			
Bisexual	2.9	1	11.1	4	7.1	5			
Asexual or unsure	23.5	8	16.7	6	20.0	14			
Pansexual	0.0	0	2.8	1	1.4	1	FFH = 6.07	.168	*V* = .30

Assigned female at birth adolescents	Early onset		Late onset		Combined		Group comparisons		
	(*n* = 203)		(*n* = 189)		(*n* = 392)		Test	*p*	Effect
Sexual orientation (in relation to birth-assigned sex)	%	*n*	%	*n*	%	*n*			
Same-sex	64.0	130	28.0	53	46.7	183			
Opposite sex	11.3	23	35.4	67	23.0	90			
Bisexual	8.9	18	19.6	37	14.0	55			
Asexual or unsure	13.8	28	13.2	25	13.5	53			
Pansexual	2.0	4	3.7	7	2.8	11	*χ*^2^(4, 392) = 61.04	<.001	*V* = .40***

This information was included at the request of a reviewer; however, as it is not the primary focus of the study, it is not discussed in further detail.

****p* < .001, FFH = Fisher–Freeman–Halton exact test.

**Table 6:** Adolescents with a late onset (*n* = 225) fulfilling the criteria for recent onset given various definitions.

DSM-5 criteria	*n*	%
Fulfilling all of the A1 to A4 criteria for less than 1 year	52	23.1
Fulfilling at least 3 of the A1 to A4 criteria for less than 1 year	63	28.0
Fulfilling at least 2 of the A1 to A4 criteria for less than 1 year	81	36.0

*DSM-5* Diagnostic and Statistical Manual of Mental Disorders, Fifth Edition.

**Table 7:** Externalizing problems and total problem score (YSR) as a function of birth-assigned sex and onset age and compared to German norm scores

	Raw scores				T scores (adolescents with GD with reference to the norm)				Clinical range (*T* scores > 63)	
	*M*	*SD*	*n*	95% CI	*M*	*SD*	*n*	95% CI	%	*n*
*Externalizing scale*										
*AMAB*										
Early onset	11.32	6.91	34	[8.91; 13.73]	53.44	7.83	34	[50.71; 56.17]	5.9	2
Late onset	11.00	5.55	36	[9.12; 12.88]	53.06	6.22	36	[50.95; 55.16]	5.6	2
Combined	11.16	6.20	70	[9.68; 12.64]	53.24	7.00	70	[51.57; 54.91]	5.7	4
*AFAB*										
Early onset	13.39	7.92	203	[12.29; 14.49]	56.49	9.16	203	[55.23; 57.76]	18.2	37
Late onset	12.40	6.81	189	[11.43; 13.38]	55.42	8.24	189	[54.24; 56.61]	13.8	26
Combined	12.91	7.41	392	[12.18; 13.65]	55.98	8.73	392	[55.11; 56.84]	16.1	63
*Total*										
Early onset	13.09	7.81	237	[12.09; 14.09]	56.05	9.03	237	[54.90; 57.21]	16.5	39
Late onset	12.18	6.63	225	[11.31; 13.05]	55.04	7.98	225	[54.00; 56.09]	12.4	28
Combined	12.65	7.26	462	[11.98; 13.31]	55.56	8.54	462	[54.78; 56.34]	14.5	67
*Total problem score*										
*AMAB*										
Early onset	49.38	21.10	34	[42.02; 56.74]	62.15	8.33	34	[59.24; 65.05]	44.1	15
Late onset	54.92	16.40	36	[49.37; 60.47]	64.47	6.32	36	[62.33; 66.61]	52.8	19
Combined	52.23	18.90	70	[47.72; 56.73]	63.34	7.41	70	[61.58; 65.11]	48.6	34
*AFAB*										
Early onset	51.74	24.29	203	[48.38; 55.11]	61.88	9.56	203	[60.55; 63.20]	39.9	81
Late onset	59.80	24.69	189	[56.26; 63.35]	65.14	9.61	189	[63.76; 66.52]	51.3	97
Combined	55.63	24.78	392	[53.17; 58.09]	63.45	9.71	392	[62.49; 64.41]	45.4	178
*Combined*										
Early onset	51.41	23.83	237	[48.36; 54.46]	61.92	9.38	237	[60.72; 63.12]	40.5	96
Late onset	59.02	23.59	225	[55.92; 62.12]	65.03	9.15	225	[63.83; 66.23]	51.6	116
Combined	55.11	24.00	462	[52.92; 57.31]	63.43	9.39	462	[62.57; 64.29]	45.9	212

Age and birth-assigned sex equivalent German norm YSR *T*-scores with *M* = 50 and *SD* = 10 were derived from Döpfner et al. (1998). The following items were excluded for the calculation of the total problem score: asthma, allergies, socially desirable items, and cross-gender identification. Raw scores for the externalizing scale range from 0 to 60, raw scores for the total problem score from 0 to 198, and *T* scores range from 25 to 100. For the clinical range, the percentage and total number of individuals scoring within the clinical range of externalizing problems/total problems are presented. These individuals scored lower than 89% of the age and birth-assigned sex equivalent reference group.

*AFAB/AMAB* assigned female/male at birth, *GD* gender dysphoria, *YSR* Youth Self-Report.

**Table 8:** Global functioning (CGAS raw scores) as a function of birth-assigned sex and onset age

	*M*	*SD*	*n*	95% CI
*AMAB*				
Early onset	66.76	12.73	34	[62.32; 71.20]
Late onset	63.89	14.60	36	[58.95; 68.83]
Combined	65.29	13.70	70	[62.02; 68.55]
*AFAB*				
Early onset	66.79	13.46	203	[64.93; 68.66]
Late onset	60.53	14.36	189	[58.47; 62.59]
Combined	63.78	14.23	392	[62.36; 65.19]
*Combined*				
Early onset	66.79	13.33	237	[65.09; 68.50]
Late onset	61.07	14.41	225	[59.17; 62.96]
Combined	64.00	14.15	462	[62.71; 65.30]

The CGAS is rated by clinicians (sum score: 1 to 100) and provides no norms (or *T* scores and clinical ranges).

*AFAB/AMAB* assigned female/male at birth, *CGAS* Children’s Global Assessment Scale, *GD* gender dysphoria.

**Table 9:** Association between externalizing problems (YSR raw scores) and the onset age

	*b*	*SE b*	95% CI for* b*	*ß*	*p*
Intercept	2.76	4.77	[−6.62; 12.13]		.564
Birth-assigned sex (0 = male, 1 = female)	2.33**	0.88	[0.59; 4.06]	.12	.009
Age in years	0.05	0.22	[−0.39; 0.49]	.01	.822
Poor peer relations (YSR)	0.85***	0.23	[0.40; 1.29]	.17	<.001
General family functioning (FAD)	4.09***	0.57	[2.96; 5.21]	.33	<.001
Body satisfaction (HBDS; *M*, *SD*)	0.24	0.41	[−0.56; 1.05]	.03	.554
Cross-gender identification (YSR; *M*, *SD*)	−0.39	0.64	[−1.64; 0.86]	−.03	.537
Sexual orientation (0 = same-sex, 1 = other)	−0.94	0.67	[−2.25; 0.36]	−.07	.157
Onset age (0 = early, 1 = late)	−1.54*	0.68	[−2.88; −0.20]	−.11	.024

**p* < .05, ***p *< .01, ****p* < .001*, *FAD = McMasters’ Family Assessment Device, *GD* gender dysphoria, *HBDS* Hamburg Body Drawing Scale, *YSR* Youth Self-Report

**Table 10:** Association between the total problem score (YSR raw scores) and the onset age

	*b*	*SE b*	95% CI for* b*	*ß*	*p*
Intercept	15.52	13.01	[−10.05; 41.08]		.234
Birth-assigned sex (0 = male, 1 = female)	7.17**	2.41	[2.44; 11.91]	.11	.003
Age in years	−0.19	0.60	[−1.37; 1.00]	−.01	.758
Poor peer relations (YSR)	5.18***	0.62	[3.96; 6.41]	.33	<.001
General family functioning (FAD)	15.19***	1.56	[12.12.; 18.26]	.38	<.001
Body satisfaction (HBDS; *M*, *SD*)	−3.59**	1.12	[−5.78; −1.39]	−.13	.001
Cross-gender identification (YSR; *M*, *SD*)	0.84	1.73	[−2.57; 4.25]	.02	.628
Sexual orientation (0 = same-sex, 1 = other)	1.91	1.81	[−1.65; 5.48]	.04	.292
Onset age (0 = early, 1 = late)	2.96	1.86	[−0.69; 6.60]	.06	.111

Results of the final model of the multiple linear regression analysis: F(8, 453) = 38.31, adjusted R2 = .39, p < .001.

**p* < .05, ***p* < .01, ****p* < .001, FAD McMasters’ Family Assessment Device, *GD* gender dysphoria, *HBDS* Hamburg Body Drawing Scale, *YSR* Youth Self-Report

**Table 11:** Association between the global functioning (CGAS) and the onset age

	*b*	*SE b*	*ß*	95% CI for* b*	*p*
Intercept	90.66***	9.54		[71.91; 109.41]	<.001
Birth-assigned sex (0 = male, 1 = female)	−2.22	1.77	−.06	[−5.70; 1.25]	.210
Age in years	−0.39	0.44	−.04	[−1.26; 0.48]	.374
Poor peer relations (YSR)	−1.72***	0.46	−.18	[−2.62; −0.83]	<.001
General family functioning (FAD)	−2.18	1.15	−.09	[−4.43; 0.07]	.058
Body satisfaction (HBDS; *M*, *SD*)	1.57	0.82	.09	[−0.04; 3.18]	.057
Cross-gender identification (YSR; *M*, SD)	−3.29*	1.27	−.12	[−5.79; −0.79]	.010
Sexual orientation (0 = same-sex, 1 = other)	−1.25	1.33	−.04	[−3.87; 1.36]	.347
Onset age (0 = early, 1 = late)	−4.75***	1.36	−.17	[−7.43; −2.08]	<.001

Results of the final model of the multiple linear regression analysis: F(8, 453) = 8.54, adjusted R2 = .12, *p* < .001

*p < .05, **p < .01, ****p* < .001, CGAS Children’s Global Assessment Scale, *FAD* McMasters’ Family Assessment Device, *HBDS* Hamburg Body Drawing Scale, *YSR* Youth Self-Report

**Table 12:** Association between internalizing problems (YSR raw scores) and the onset age (late and recent onset age separated)

	*b*	*SE b*	*ß*	95% CI for* b*	*p*
Intercept	6.75	5.90		[−4.85; 18.35]	.254
Birth-assigned sex (0 = male, 1 = female)	2.67*	1.09	.09	[0.52; 4.81]	.015
Age in years	−0.07	0.27	−.01	[−0.61; 0.47]	.793
Poor peer relations (YSR)	2.68***	0.28	.36	[2.13; 3.24]	<.001
General family functioning (FAD)	5.94***	0.71	.32	[4.55; 7.33]	<.001
Body satisfaction (HBDS; *M*, *SD*)	−2.67***	0.51	−.20	[−3.67; −1.68]	<.001
Cross-gender identification (YSR; *M*, *SD*)	0.55	0.79	.03	[−0.99; 2.09]	.485
Sexual orientation (0 = same-sex, 1 = other)	2.20**	0.82	.10	[0.59; 3.82]	.008
Late onset age (= 1, early = 0)	3.15***	0.90	.14	[1.39; 4.91]	<.001
Recent onset age (= 1, early = 0)	2.51	1.31	.07	[−0.06; 5.08]	.055

**p *< .05, ***p *< .01, ****p* < .001*, **CGAS* Children’s Global Assessment Scale, *FAD* McMasters’ Family Assessment Device, *YSR* Youth Self-Report

## Footnotes

1 It is important to note that these observations are based on the clinical experience of our gender identity service and that, at present, no scientific evidence exists to substantiate them.
